# A novel Online-to-Offline (O2O) model for pre-exposure prophylaxis and HIV testing scale up

**DOI:** 10.7448/IAS.20.1.21326

**Published:** 2017-03-10

**Authors:** Tarandeep Anand, Chattiya Nitpolprasert, Deondara Trachunthong, Stephen J Kerr, Surang Janyam, Danai Linjongrat, Lisa B Hightow-Weidman, Praphan Phanuphak, Jintanat Ananworanich, Nittaya Phanuphak

**Affiliations:** ^a^ The Thai Red Cross AIDS Research Centre, Bangkok, Thailand; ^b^ SEARCH, The Thai Red Cross AIDS Research Centre, Bangkok, Thailand; ^c^ HIV-NAT, The Thai Red Cross AIDS Research Centre, Bangkok, Thailand; ^d^ Department of Global Health, Academic Medical Center, University of Amsterdam, Amsterdam Institute for Global Health and Development, Amsterdam, The Netherlands; ^e^ The Kirby Institute, University of New South Wales, Sydney, Australia; ^f^ Service Workers in Group Foundation, Bangkok, Thailand; ^g^ Rainbow Sky Association of Thailand, Bangkok, Thailand; ^h^ Division of Infectious Diseases, School of Medicine, University of North Carolina at Chapel Hill, Chapel Hill, NC, USA; ^i^ US Military HIV Research Program, Walter Reed Army Institute of Research, Silver Spring, MD, USA; ^j^ Henry M. Jackson Foundation for the Advancement of Military Medicine, Bethesda, MD, USA

**Keywords:** pre-exposure prophylaxis, PrEP scale up, PrEP uptake, Thailand, MSM, transgender women, technology, innovative model

## Abstract

**Introduction**: PrEP awareness and uptake among men who have sex with men (MSM) and transgender women (TG) in Thailand remains low. Finding ways to increase HIV testing and PrEP uptake among high-risk groups is a critical priority. This study evaluates the effect of a novel Adam’s Love Online-to-Offline (O2O) model on PrEP and HIV testing uptake among Thai MSM and TG and identifies factors associated with PrEP uptake.

**Methods**: The O2O model was piloted by Adam’s Love (www.adamslove.org) HIV educational and counselling website. MSM and TG reached online by PrEP promotions and interested in free PrEP and/or HIV testing services contacted Adam’s Love online staff, received real-time PrEP eCounseling, and completed online bookings for receiving services at one of the four sites in Bangkok based on their preference. Auto-generated site- and service-specific e-tickets and Quick Response (QR) codes were sent to their mobile devices enabling monitoring and check-in by offline site staff. Service uptake and participant’s socio-demographic and risk behaviour characteristics were analyzed. Factors associated with PrEP uptake were assessed using multiple logistic regression.

**Results**: Between January 10th and April 11th, 2016, Adam’s Love reached 272,568 people online via the PrEP O2O promotions. 425 MSM and TG received eCounseling and e-tickets. There were 325 (76.5%) MSM and TG who checked-in at clinics and received HIV testing. Nine (2.8%) were diagnosed with HIV infection. Median (IQR) time between receiving the e-ticket and checking-in was 3 (0–7) days. Of 316 HIV-negative MSM and TG, 168 (53.2%) started PrEP. In a multivariate model, higher education (OR 2.30, 95%CI 1.14–4.66; *p* = 0.02), seeking sex partners online (OR 2.05, 95%CI 1.19–3.54; *p* = 0.009), being aware of sexual partners’ HIV status (OR 2.37, 95%CI 1.29–4.35; *p* = 0.008), ever previously using post-exposure prophylaxis (PEP) (OR 2.46, 95%CI 1.19–5.09; *p* = 0.01), and enrolment at Adam’s Love clinic compared to the other three sites (OR 3.79, 95%CI 2.06–6.95; *p* < 0.001) were independently associated with PrEP uptake.

**Conclusions**: Adam’s Love O2O model is highly effective in linking online at-risk MSM and TG to PrEP and HIV testing services, and has high potential to be replicated and scaled up in other settings with high Internet penetration among key populations.

## Introduction

Thailand’s explosive HIV epidemic among men who have sex with men (MSM) and transgender women (TG) remains a major public health concern. In Bangkok, one in every three MSM are estimated to be HIV-positive [1] and HIV incidence rates, especially among young MSM (YMSM) aged 18–21 years, are alarmingly high (12.2 per 100 person years) [2]. Prevalence estimates from studies on Thai TG range from 10% to 17% [3–5]. Thailand aims to end the AIDS epidemic by 2030 and modelling showed the only way to achieve this goal is to increase HIV testing to cover 90% of key populations, to treat all HIV cases with antiretroviral therapy (ART) regardless of CD4 count [6], and to harness and maximize the use of innovative biomedical HIV prevention tools, such as pre-exposure prophylaxis (PrEP) for most-at risk populations.

PrEP, with daily use of a fixed-dose combination tablet of tenofovir disoproxil fumarate (TDF) and emtricitabine (FTC) is a safe, effective method of preventing HIV among MSM and TG [7–11]. The WHO Consolidated guidelines on HIV testing, treatment, and prevention call for expanded access to PrEP for all individuals who are at substantial ongoing risk of acquiring HIV, provisionally defined as an incidence of HIV greater than three per 100 person-years [12]. Given incidence rates and sexual and drug use risk behaviours among Thai MSM and TG [2,13], increasing uptake of PrEP is a critical priority.

Thailand was one of the first Asian countries to participate in PrEP clinical trials and implementation projects [14–17]. A number of demonstration projects are currently underway. To date, Thailand has relied heavily on traditional outreach models for PrEP scale up and delivery, despite inherent challenges with engaging hard to reach and closeted MSM [17,18]. A recent programme conducted under the Thailand Global Fund for AIDS, Tuberculosis and Malaria found that only 7% of MSM reached through offline outreach received HIV testing [19], a critical first step to confirm an HIV-negative status prior to initiation of PrEP and reduce the risk of drug resistance. The rate of annual HIV testing among the Thai MSM population, which is estimated to contribute to 40% of country’s new HIV cases during 2012–2016, is also very low at merely 29% [20,21]. HIV counselling and testing is a point of engagement into the HIV prevention continuum offering providers’ opportunities to foster bi-directional communication with clients, assess risks, introduce PrEP as one method in the combination HIV prevention package and encourage uptake. Lack of HIV testing and repeat testing of those at high risk of HIV infection remains the first and most critical barrier to PrEP awareness and subsequent PrEP uptake.

There is limited data on PrEP awareness among Thai MSM and TG communities and among those who are aware of PrEP multiple barriers to PrEP uptake still exist. In a study conducted in 2013 by Sineath et al., only few respondents (7%) reported having heard of PrEP [22]. However, Thai MSM have indicated a willingness to take PrEP, even if they had to experience inconvenience and expense [23]. Thus, finding ways to identify those individuals at highest risk through innovative and scalable testing methodologies, and seamlessly transitioning them into biomedical prevention services is needed. Particularly, extending outreach to include online venues is vital considering that Thai MSM and TG have some of the highest internet and technology utilization [24].

### Adam’s Love website

Adam’s Love (www.adamslove.org), a technology-based HIV outreach, counselling and testing initiative in Asia for MSM and TG individuals, was launched in September 2011 by the Thai Red Cross AIDS Research Centre (TRCARC) and has demonstrated success in scaling-up HIV testing in Thailand and Indonesia [25–27]. Adam’s Love comprises of an HIV educational website, eCounseling platforms and integrated social media networks. Since its launch, Adam’s Love engaged more than 2.8 million website visitors. An estimated 17,357 MSM and TG individuals received real-time counselling at Adam’s Love eCounseling platforms and were successfully linked to relevant clinical services, for example, HIV and sexually transmitted infection (STI) testing, treatment, and care and post-exposure prophylaxis (PEP) [28]. Of MSM and TG clients who annually received HIV testing at TRCARC, an estimated 25% were recruited online via Adam’s Love and an HIV prevalence of 15.5% was reported in 2015 [25].

### Gap that emerged in terms of connecting online visitors with critical offline clinical services

Previous efforts by Adam’s Love included online HIV testing and MSM/TG health promotions, eCounseling support and linkage to relevant clinical services, with limited focus on PrEP. Additionally, MSM and TG eCounseled and linked to offline sites had to start over the process of explaining their risk behaviours and service preference to the clinic reception staff. This often led to reluctance, confidentially concerns and fragmented experiences.

Our study evaluates the effect of a novel Online-to-Offline (O2O) model, piloted by Adam’s Love (www.adamslove.org) HIV educational and counselling website, on PrEP and HIV testing uptake among Thai MSM and TG.

## Methods

### Adam’s Love O2O model

Adam’s Love O2O model included three key features:

(a) tailored social media PrEP promotions including PrEP educational content, and photos/videos of Thai male celebrities and MSM and TG community promoting Adam’s Love non-financial incentives (i.e. bags, caps and T-shirts) for individuals receiving free PrEP and/or HIV testing services at clinic sites.

(b) refined eCounseling intervention to provide PrEP eCounseling support on Adam’s Love platforms (i.e. web message boards, popular social media and instant messaging applications with text and live video chat, and media sharing capabilities) and assess the risks of those eCounseled by asking a set of standardized questions (Appendix 1) including age; ever having an anal intercourse; number of sexual partners, condom use during anal sex and HIV testing in the past six months; and date of last possible HIV risk exposure.

(c) a free online booking system to facilitate effective linkage to relevant offline clinical services and a real-time monitoring feature to track individuals successfully linked to sites.

**Figure 1.**
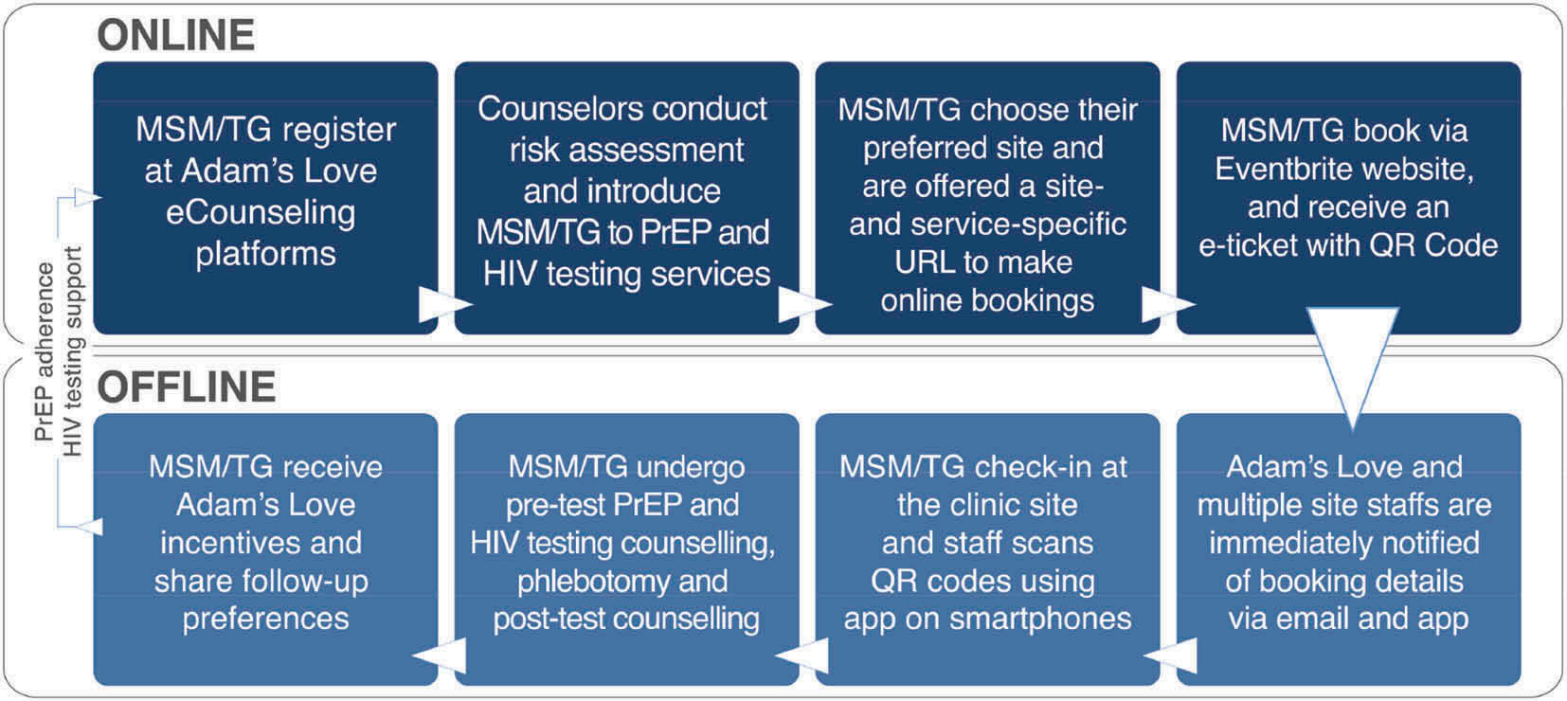
The Adam’s Love Online-to-Offline (O2O) model for PrEP and HIV testing scale up. The O2O model includes eight steps: (1) MSM and TG people register at Adam’s Love eCounseling platforms and contact online counsellors, (2) Adam’s Love online counsellors conduct an individualized risk assessment by asking a set of standardized questions (consistent for all participants), introduce them to relevant PrEP and HIV testing services and enrol them in the study, (3) participants then prioritize their preferred site for receiving service and are offered a site- and service-specific booking URL to make online bookings, (4) participants register for free via Eventbrite (EVB) website, chose their preferred date and check-in time for their specified service and receive an email confirmation with an e-ticket with a QR code confirming the time and date of the booking, and a reminder email one day prior to the check-in date, (5) Adam’s Love site staff are immediately notified of the booking details via email and through the EVB app once a booking is made, (6) participants check-in at the clinic site and staff scans the QR codes (print or digital) on tickets using smartphone devices or manually via electronic lists, and a green bar appears next to the attendee’s order indicating that they’ve been successfully checked in, (7) participants undergo pre-test HIV testing counselling, phlebotomy and post-test counselling. If negative, PrEP is provided and (8) participants receive Adam’s Love merchandise (e.g. t-shirts, caps, tote bags), and share their preferred online platform, content and frequency for adherence or testing reminders.

In January 2016, Adam’s Love set out to explore the potential of its O2O model (Figure 1) for PrEP scale up among Thai MSM and TG to test and ascertain the most appropriate approaches to the expansion of PrEP services in Thailand and other countries with widespread internet access and an educated population.

### Clinical partners

Adam’s Love launched a three-month pilot programme to enrol MSM and TG into free PrEP and HIV testing services at four sites in Bangkok including TRCARC Anonymous Clinic, Adam’s Love Clinic (an MSM and TG-focused and by-online-appointment only clinic operated by TRCARC) and two community-based drop-in centres including Rainbow Sky Association of Thailand (RSAT) which targeted MSM and TG and Service Workers In Group (SWING) Foundation which targeted male sex workers. The two drop-in centres provided PrEP as part of “community-led HIV services” run solely by non-medical community staff, who were trained and coached to perform counselling, rapid HIV and syphilis testing, sample collection for STI screening, PEP and PrEP prescription, case management, and adherence and retention support for both HIV-positive clients and HIV-negative clients. The non-medical community staff received ongoing quality assessment and quality improvement by the TRCARC. PrEP medication was free for participants in this programme through the Thai Red Cross Princess Soamsawali Prevention Fund.

### Pilot test of O2O

MSM and TG reached online by O2O PrEP social media promotions (Supplementary Figure 1) and interested in free PrEP and/or HIV testing services contacted Adam’s Love online staff, received real-time PrEP eCounseling, were assessed for risks through a set of standardized questions (Appendix 1) and enrolled at one of the four sites in Bangkok based on their preference. Participants prioritized their preferred service and clinic site during the eCounseling session and were provided a site- and service-specific booking URL. Participants made free online bookings via the Eventbrite (EVB) application (app) – www.eventbrite.com, a self-service ticketing platform and received an auto-generated email confirming the booking summary, a downloadable and a Portable Document Format (PDF) attachment of an e-ticket with booking details, maps and directions to the clinic site, barcodes and a Quick Response (QR) code. The codes were later scanned using the EVB Organizer mobile app at check-in by offline site staff using smartphone devices. Adam’s Love and site staff would receive an e-ticket order number in an email each time a booking was made, and could track check-in data in real time and monitor live check-in statistics from the “Dashboard” within the app. A timestamp captured the time gap between placing the online order and check-in date at the offline site (Figure 1).

Data collected at the clinic site through participant-completed online questionnaires included socio-demographic and risk behaviour data such as age, education, income, sexual identity, social media usage and online sex seeking behaviour, HIV/STI test history, condom use, number of sexual partners and drug use (including alcohol, popper, ecstasy, cannabis, cocaine, methamphetamine, ketamine, LSD, and heroine) in the past 6 months. Online metrics included page views, reach (number of people who saw the promotions), number of orders, daily tickets ordered, attendee geolocation, order and attendance time, no-show rates and clinic site attended. Clinical and laboratory data included HIV testing and PrEP uptake and HIV test results.

All participants in the O2O programme were designated male at birth, reported having had anal intercourse with men, and were aged 18 years or above. Consent was provided online. Participants who tested positive for HIV were not eligible for PrEP. The study protocol was approved by the Institutional Review Board (IRB) of the Faculty of Medicine, Chulalongkorn University in Bangkok, Thailand.

### Statistical analysis

The total number of people reached through O2O PrEP promotions was measured using web and social media analytics tools including YouTube (www.youtube.com) analytics and Facebook (www.facebook.com) page insights.

Statistical analysis was conducted with Stata 14 (StataCorp LP, College Station, TX, USA). Descriptive statistics were used to summarize mean (SD), median (IQR) for continuous variables; frequency and percentage for categorical variables. Site characteristics were compared using a one-way ANOVA or Kruskal–Wallis test for continuous variables and categorical variables were tested a Chi-squared test or Fisher’s exact test, as appropriate. Multiple logistic regression was used to identify factors associated with the uptake of PrEP among all HIV-negative participants who made their visit. Covariates modelled included programme details such as time interval between ordering the e-ticket and actual check in, socio-demographics, patterns of social media use, self-reported risk behaviour and history of STIs. Factors significant at *p* ≤ 0.1 in univariate analysis were adjusted for in a multivariate model.

## Results

Between January 10th and April 11th, 2016, Adam’s Love reached 272,568 people online via PrEP O2O social media promotions. 425 MSM and TG received eCounseling, made online bookings, and received QR codes and e-tickets. There were 325 (76.5%) MSM and TG who checked-in at one of the four study clinics and received HIV testing, Nine (2.8%) were diagnosed with HIV infection. Median age of participants was 27 years (IQR: 23–33), most (77.2%) had at least a high school education, majority (61.8%) had monthly income above 500 USD, most (77.9%) self-identified as gay and more than half (56.9%) hid their sexual identity. Almost a quarter of the participants (22.8%) had more than five sexual partners in the past six months, 26.8% reported ever having had an STI, almost half (48.1%) had sometimes or never used condoms in the past six months and less than one-third (30.2%) were aware of sexual partners HIV status. More than one-third of the participants (35.4%) had used drugs in the past six months of whom almost a quarter (21.7%) were amphetamine-type stimulants (ATS) users. Almost half (46.8%) of the participants were aware of PEP as an HIV prevention method of whom more than one-third (34.2%) had used PEP in the past. The majority (52.6%) spent more than seven hours per day using social media and almost one-third (32.6%) reported having sought sexual partners online.

Median (IQR) time between receiving e-ticket and check-in was 3 (0–7) days. Most participants (44%) checked in and received services at TRCARC Anonymous Clinic, one-third (34.1%) at Adam’s Love clinic, and the rest (21.9%) at the two community-based clinics. PrEP uptake was highest (70.4%) at Adam’s Love clinic compared with 46.8% at TRCARC Anonymous Clinic and 39.1% at community-based clinics (*p* < 0.001). Median age (IQR), having ever been tested for HIV, STI rates, social media usage and online sex seeking behaviour were similar among participants at all sites. Participants at TRCARC Anonymous Clinic and Adam’s Love clinic were higher educated (*p* < 0.001), had higher income (*p* < 0.001), were less likely to have lifetime sexual relationship with both male and female (*p* = 0.004) or currently be in relationship with both male and female (*p* = 0.006) and had fewer sexual partners in the past six months (*p* = 0.03) than those at the community-based clinics. Adam’s Love and community-based clinic participants were more likely to be based in Bangkok (*p* < 0.001), less likely to be aware of their sexual partner’s HIV status (*p* = 0.006) and less likely to have received an HIV test in the past six months (*p* = 0.001) than TRCARC Anonymous Clinic participants. Although not statistically significant, drug use behaviour was highest (45%) among participants at community-based clinics (Table 1).

**Table 1.** Descriptive characteristics of all participants

Characteristics *N* (%)	All sites (*N* = 325)	Adam’s Love clinic (*N* = 111)	TRCARC clinic (*N* = 143)	Community-based sites (*N* = 71)	*p*-Value
**Days between ticket and check-in**					
Median (IQR)	3 (0–7)	4 (2–8)	4 (1–8)	0 (0–0)	<0.001^b^
Min, Max	0, 45	0, 33	0, 45	0, 21	
**Monthly check-in (visits)^e^**					<0.001^c^
Month 1	77 (23.7)	27 (24.3)	40 (28.0)	10 (14.1)	
Month 2	94 (28.9)	16 (14.4)	41 (28.7)	37 (52.1)	
Month 3	154 (47.4)	68 (61.3)	62 (43.3)	24 (33.8)	
**HIV test result**					>0.99^d^
Negative	316 (97.2)	108 (97.3)	139 (97.2)	69 (97.2)	
Positive	9 (2.8)	3 (2.7)	4 (2.8)	2 (2.8)	
**PrEP uptake among HIV-negative participants**					<0.001^c^
Non-PrEP users (HIV testers)	148 (46.8)	32 (29.6)	74 (53.2)	42 (60.9)	
PrEP users	168 (53.2)	76 (70.4)	65 (46.8)	27 (39.1)	
**Age (years)**					
Mean (SD)	28.5 (7.4)	28.6 (7.5)	27.8 (7.2)	29.8 (7.8)	0.19^a^
Median (IQR)	27 (23–33)	27 (23–34)	27 (23–32)	28 (24–34)	
**Age group**					0.84^c^
≤18	16 (4.9)	6 (5.4)	8 (5.6)	2 (2.8)	
19–24	93 (28.6)	31 (27.9)	45 (31.5)	17 (23.9)	
25–34	150 (46.2)	52 (46.9)	63 (44.1)	35 (49.3)	
>34	66 (20.3)	22 (19.8)	27 (18.9)	17 (23.9)	
**Current residence**					<0.001^c^
Outside Bangkok	37 (11.4)	7 (6.3)	29 (20.3)	1 (1.4)	
Bangkok Metropolitan Region	288 (88.6)	104 (93.7)	114 (79.7)	70 (98.6)	
**Education level**					<0.001^c^
High school or below	74 (22.8)	15 (13.5)	18 (12.6)	41 (57.8)	
Above High school	251 (77.2)	96 (86.5)	125 (87.4)	30 (42.3)	
**Monthly income**					<0.001^c^
No salary as studying	59 (18.2)	16 (14.4)	36 (25.2)	7 (9.9)	
No salary as unemployed	15 (4.6)	0 (0)	7 (4.9)	8 (11.3)	
≤500 USD	50 (15.4)	10 (9)	14 (9.8)	26 (36.6)	
501–1,000 USD	132 (40.6)	48 (43.2)	58 (40.6)	26 (36.6)	
>1,000 USD	69 (21.2)	37 (33.3)	28 (19.6)	4 (5.6)	
**Self-identified sexuality**					<0.001^c^
Gay	253 (77.9)	97 (87.4)	115 (80.4)	41 (57.8)	
Bisexual	39 (12)	10 (9)	18 (12.6)	11 (15.5)	
Transgender	17 (5.2)	2 (1.8)	7 (4.9)	8 (11.3)	
Male	16 (4.9)	2 (1.8)	3 (2.1)	11 (15.5)	
**Disclosure status of gender identity**					0.58^c^
Discreet (hidden)	63 (19.4)	21 (18.9)	27 (18.9)	15 (21.1)	
Open to some	122 (37.5)	42 (37.8)	59 (41.3)	21 (29.6)	
Open to all (out)	140 (43.1)	48 (43.2)	57 (39.9)	35 (49.3)	
**Lifetime sexual relationships with which genders?**					0.004^d^
Male only	233 (71.7)	89 (80.2)	104 (72.7)	40 (56.3)	
Male and female	81 (24.9)	20 (18)	33 (23.1)	28 (39.4)	
Female and TG	5 (1.5)	2 (1.8)	3 (2.1)	0 (0)	
Male and TG	1 (0.3)	0 (0)	1 (0.7)	0 (0)	
All (male, female, TG)	5 (1.5)	0 (0)	2 (1.4)	3 (4.2)	
**Current sexual relationships with which genders?**					0.006^d^
Male only	277 (85.2)	101 (91)	125 (87.4)	51 (71.8)	
Female only	8 (2.5)	1 (0.9)	4 (2.8)	3 (4.2)	
TG	3 (0.9)	0 (0)	2 (1.4)	1 (1.4)	
Male and female	32 (9.9)	7 (6.3)	11 (7.7)	14 (19.7)	
Female and TG	3 (0.9)	2 (1.8)	1 (0.7)	0 (0)	
All (male, female, TG)	2 (0.6)	0 (0)	0 (0)	2 (2.8)	
**Ever tested for HIV**					0.80^c^
No	59 (18.2)	21 (18.9)	27 (18.9)	11 (15.5)	
Yes	266 (81.9)	90 (81.1)	116 (81.1)	60 (84.5)	
**Previous HIV test**					0.001^c^
≤1 month	92 (28.3)	18 (16.2)	58 (40.6)	16 (22.5)	
1–6 months	53 (16.3)	24 (21.6)	15 (10.5)	14 (19.7)	
6 months–1 year	84 (25.9)	38 (34.2)	26 (18.2)	20 (28.2)	
>1 year	31 (9.5)	10 (9)	14 (9.8)	7 (9.9)	
Never tested for HIV/can’t remember	65 (20)	21 (18.9)	30 (21)	14 (19.7)	
**Ever had an STI**					0.57^c^
No	208 (64)	67 (60.4)	96 (67.1)	45 (63.4)	
Yes	87 (26.8)	34 (30.6)	32 (22.4)	21 (29.6)	
Don’t know/never tested	30 (9.2)	10 (9)	15 (10.5)	5 (7)	
**Time using social media per day**					0.19^c^
1–3 h a day	44 (13.5)	11 (9.9)	19 (13.3)	14 (19.7)	
3–7 h a day	110 (33.9)	33 (29.7)	53 (37.1)	24 (33.8)	
>7 h a day	171 (52.6)	67 (60.4)	71 (49.7)	33 (46.5)	
**Ever seek sexual partners online**					0.46^c^
No	219 (67.4)	78 (70.3)	98 (68.5)	43 (60.6)	
Yes	106 (32.6)	33 (29.7)	45 (31.5)	28 (39.4)	
**Number of sexual partners in the past 6 months**					0.03^d^
Didn’t have sex in past 6 months	10 (3.1)	3 (2.7)	5 (3.5)	2 (2.8)	
One	83 (25.5)	26 (23.4)	42 (29.4)	15 (21.1)	
2–5	158 (48.6)	65 (58.6)	66 (46.2)	27 (38)	
5–10	38 (11.7)	11 (9.9)	14 (9.8)	13 (18.3)	
>10	36 (11.1)	6 (5.4)	16 (11.2)	14 (19.7)	
**Condom use in the past 6 months**					0.48^d^
Didn’t have sex in past 6 months/refused to answer	13 (4)	3 (2.7)	8 (5.6)	2 (2.8)	
Always	156 (48)	49 (44.1)	66 (46.2)	41 (57.8)	
Sometimes	137 (42.2)	51 (46)	60 (42)	26 (36.6)	
Never	19 (5.9)	8 (7.2)	9 (6.3)	2 (2.8)	
**Sexual partners’ HIV status awareness**					0.006^c^
No	167 (51.4)	61 (55)	62 (43.4)	44 (62)	
Yes	98 (30.2)	25 (22.5)	58 (40.6)	15 (21.1)	
Never ask	60 (18.5)	25 (22.5)	23 (16.1)	12 (16.9)	
**Substance use in past 6 months (including alcohol)**					0.11^c^
No	210 (64.6)	78 (70.3)	93 (65)	39 (54.9)	
Yes	115 (35.4)	33 (29.7)	50 (35)	32 (45.1)	
**Amphetamine-type stimulants**					0.06^c^
No	300 (92.3)	106 (95.5)	133 (93)	61 (85.9)	
Yes	25 (7.7)	5 (4.5)	10 (7)	10 (14.1)	
**Aware of PEP as an HIV prevention method**					0.07^c^
No	173 (53.2)	50 (45.1)	85 (59.4)	38 (53.5)	
Yes	152 (46.8)	61 (55)	58 (40.6)	33 (46.5)	
**Ever used PEP**					0.80^c^
No	273 (84)	95 (85.6)	118 (82.5)	60 (84.5)	
Yes	52 (16)	16 (14.4)	25 (17.5)	11 (15.5)	

^a^One-way ANOVA. ^b^Kruskal–Wallis. ^c^Chi-square test. ^d^Fisher’s exact test. ^e^Monthly check-in refers to the number of participants checked in and accessing HIV testing and PrEP services in each month.

Of 316 HIV-negative MSM and TG, 168 (53.2%) decided to start PrEP. In a multivariate model adjusting for income and ever having tested for HIV, higher education (OR 2.30, 95%CI 1.14–4.66; *p* = 0.02), seeking sex partners online (OR 2.05, 95%CI 1.19–3.54; *p* = 0.009), being aware of sexual partners’ HIV status (OR 2.37, 95%CI 1.29–4.35; *p* = 0.008), ever previously using PEP (OR 2.46, 95%CI 1.19–5.09; *p* = 0.01), and enrolment at Adam’s Love clinic (OR 3.79, 95%CI 2.06–6.95; *p* < 0.001) were independently associated with PrEP uptake. Past STI history, anal sex role, condom use, number of sexual partners and drug use in the past 6 months were not associated with PrEP uptake (Table 2).

**Table 2.** Logistic regression model of factors associated with using PrEP among all HIV-negative participants who made their visit. (Factors significant at *p* ≤ 0.1 in univariate analysis were adjusted for in a multivariate model)

	Non-PrEP users (*n* = 148)		PrEP users (*n* = 168)		Univariate			Multivariate		
Characteristics	freq	%	freq	%	OR	95%C.I.	*p*-Value	OR	95%C.I.	*p*-Value
**Days between ticket and check-in**							0.56			
<4	85	57%	91	54%	1	ref				
≥4	63	43%	77	46%	1.14	0.73–1.78				
**Service delivery site**							<0.001			<0.001
TRCARC clinic	74	50%	65	39%	1	ref		1	ref	
Adam’s Love clinic	32	22%	76	45%	2.70	1.59–4.60		3.79	2.06–6.95	
Community-based sites	42	28%	27	16%	0.73	0.41–1.32		1.13	0.54–2.33	
**Education level**							<0.001			0.02
High school or below	49	33%	23	14%	1	ref		1	ref	
Above High school	99	67%	145	86%	3.12	1.79–5.45		2.30	1.14–4.66	
**Monthly income**							0.003			0.45
No salary as studying/unemployed	43	29%	30	18%	1	ref		1	ref	
≤500 USD	29	20%	18	11%	0.89	0.42–1.88		1.10	0.47–2.61	
501–1000 USD	50	34%	79	47%	2.26	1.26–4.07		1.43	0.74–2.76	
>1000 USD	26	18%	41	24%	2.26	1.15–4.45		0.85	0.39–1.87	
**Ever tested for HIV**							0.01			>0.99
No	35	24%	22	13%	1	ref		1	ref	
Yes	113	76%	146	87%	2.05	1.14–3.70		1.002	0.42–2.39	
**Ever had any STI**							0.40			
No	99	67%	106	63%	1	ref				
Yes	34	23%	49	29%	1.35	0.80–2.26				
Don’t know/never tested	15	10%	13	8%	0.81	0.37–1.79				
**Ever seek sexual partners online**							0.02			0.009
No	109	74%	103	61%	1	ref		1	ref	
Yes	39	26%	65	39%	1.76	1.09–2.85		2.05	1.19–3.54	
**Number of sexual partners in the past 6 months**							0.60			
0–1	44	30%	46	27%	1	ref				
2–5	72	49%	80	48%	1.06	0.63–1.79				
5–10	19	13%	19	11%	0.96	0.45–2.04				
>10	13	9%	23	14%	1.69	0.76–3.75				
**Anal sex role**							0.78			
Insertive	52	35%	67	40%	1	ref				
Receptive	39	26%	45	27%	0.89	0.51–1.57				
Both	48	32%	47	28%	0.76	0.44–1.30				
Didn’t have sex	9	6%	9	5%	0.78	0.29–2.09				
**Condom use in the past 6 months**							0.50			
Always/didn’t have sex	73	49%	91	54%	1	ref				
Sometimes	64	43%	69	41%	0.86	0.55–1.37				
No	11	7%	8	5%	0.58	0.22–1.52				
**Sexual partners’ HIV status awareness**							0.004			0.008
No	81	55%	81	48%	1	ref		1	ref	
Yes	33	22%	64	38%	1.94	1.15–3.26		2.37	1.29–4.35	
Never ask	34	23%	23	14%	0.68	0.37–1.25		0.77	0.38–1.52	
**Substance use in past 6 months (including alcohol)**							0.28			
No	101	68%	105	63%	1	ref				
Yes	47	32%	63	38%	1.29	0.81–2.06				
**Ever used PEP**							<0.001			0.01
No	135	91%	129	77%	1	ref		1	ref	
Yes	13	9%	39	23%	3.14	1.6–6.15		2.46	1.19–5.09	

## Discussions

Previously conducted studies have mostly focused on PrEP awareness, PrEP acceptability, and willingness among MSM and TG groups and the barriers and facilitators [29]. We demonstrated for the first time that a novel O2O model could reach high-risk, closeted MSM and TG individuals and successfully bridge online outreach to offline uptake of HIV testing and PrEP services.

Greater attention should be placed on increasing PrEP awareness through public health campaigns targeting MSM with high-risk behaviours [30]. In our study, tailored social media promotions successfully reached a large number of Thai MSM and TG groups, a critical challenge faced by traditional and one-on-one outreach models. Of the large number of people who saw the promotions, 0.16% actively contacted counsellors and engaged in eCounseling. Participation in social media implies an excess effort to act on the promotional post compared with seeing the post and could have been low due to multiple factors such as content type, day/timing of promotion or divided attention [31,32]. It is also possible that only motivated individuals contemplating behaviour change [33] or seeking new HIV prevention methods participated in eCounseling. Access to and capacity of eCounseling were not determining factors in limited uptake as all possible efforts were made to ensure prompt responses and establish dialogue same day an inquiry was made.

The community-based sites staff with Internet-enabled and EVB app-installed smartphones promoted free PrEP and encouraged online bookings as part of their daily face-to-face outreach activities in various hotspot areas, leading to mostly same day check-ins at community-based sites. Internet access or literacy was not reported as a challenge by either staff or client. More efforts and trainings to strengthen community-based site staffs’ online outreach and scheduling capabilities are needed.

Some MSM self-identify as straight male, especially if they also have sex with women, are married, take insertive role in anal sex [34], and/or have sex with men for money [35]. This identity was commonly selected at community-based sites with the highest number of discreet participants. It is also possible that these included low income straight-identified MSM who engaged in transactional sex.

Several studies have found that one of the biggest barriers to the provision of PrEP are the reticence of healthcare providers [36–39] and suggested that PrEP programmes need to be coupled with HIV risk management counselling in stigma-free settings [40]. Real-time eCounseling and support by Adam’s Love experienced and trained counsellors helped foster relationships with MSM and TG individuals reached online, assess individuals at risk for HIV infection, provide basic education about PrEP and how to access it in virtual non-judgmental settings. This may have helped reduce social, structural and individual-level barriers before enrolling them into actual clinical services. Online booking process involved entering a preferred pseudonym and a valid email address. No additional personal identifying information was needed to enter the programme and helped overcome participant confidentiality and privacy concerns, a critical barrier to accessing sexual health services among Thai MSM and TG [41]. Use of technology with real-time monitoring features helped track, validate participants and identify participants’ choice of service. Such innovative monitoring approaches could help fill significant programmatic gaps in tracking individuals through Thailand’s Reach-Recruit-Test-Treat-Retain cascade [42].

A majority of those who made online bookings and received e-tickets (76.4%), actually checked in to receive free PrEP and HIV testing services. High PrEP uptake has been reported when made available free of charge by experienced providers [10], possibly contributing to high interest and uptake among MSM and TG participants in our study.

The clinic site itself played a significant role in participant’s preference for PrEP uptake. A private and friendly by-appointment clinic was preferred over public clinic and community-based sites for enrolling in PrEP service, this pattern may be due to multiple stigmas associated with PrEP among MSM and TG communities including stigma of being related to HIV and other stigmas, such as homosexuality, sex work, and/or drug use [43], suggesting higher confidentiality and anonymity needs for those interested in enrolling in PrEP services. Although staffing and other resources differed across the four clinic sites, we believe that counselling at the site did not impact PrEP uptake as all participants had prioritized their preferred service during eCounseling session and approach was consistent. Extending online promotions and outreach through existing social media networks of community-based sites staff and popular online platforms used for seeking sex are critical to reach, engage and scale up PrEP among high-risk groups in the future.

Similar to the results of the PrEP Brasil demonstration project [44], most MSM and TG individuals enrolled in PrEP in our study were young adults (age 26–35 years). A higher level of education is related to an increased likelihood of taking PrEP [45], our PrEP participants had higher education and higher income, which may indicate a greater understanding of the medication or greater overall health literacy and greater awareness of partner’s HIV status. It may also be possible that some participants were in serodiscordant relationships and were planning to use PrEP with their HIV-positive partners.

Those who had sought sex partners on the Internet and those with past PEP use were more likely to choose and receive PrEP services. Prior PEP use was found to be a predictor of interest and willingness in taking PrEP among MSM who engage in online networking [46]. Self-identifying as bisexual has been previously associated with increased PrEP awareness [46]. However, PrEP users in our study were less likely to be bisexual, this might be because of the stigma associated with bisexuality and less willingness among Thai bisexual MSM to seek clinical services [47].

Our results demonstrate that PrEP uptake was unrelated with risk behaviours including drug use, condom-use behaviour, number of sexual partners and STI history, in contrast to previously published studies that have found PrEP acceptability to be correlated with higher-risk behaviours [46,48–50]. More efforts are therefore needed to build self-risk assessment abilities and encourage PrEP uptake among these vulnerable MSM and TG groups, a significant consideration for the next generation of Adam’s Love O2O platform. Further, the O2O model was less successful at engaging MSM and TG with lower education to PrEP services, indicating future tailoring of the model for wider PrEP scale up.

## Limitations

To participate in our O2O programme, participants had to have access to a computer- or a web-enabled mobile device such as a tablet or smartphone. Given high internet and technology utilization among Thai MSM and TG [24], we assume that this limitation would not have been a major barrier to participation. Another limitation is that we did not focus on tailoring our intervention to support and measure PrEP adherence, a critical priority to ensure PrEP efficacy and offset the development of resistance. This remains the key focus for the next phase of O2O programme.

## Conclusions

Our study demonstrates that Adam’s Love O2O model is highly effective in linking online at-risk MSM and TG to PrEP and HIV testing services, using eCounseling and booking as ‘bridging steps’ likely by overcoming the barriers and challenges to PrEP uptake. The study reveals significantly high interest in PrEP uptake among Thai MSM and TG and helps identify predictors of PrEP uptake. The O2O model has high potential to be replicated and scaled up in other settings with high internet penetration among key populations, where MSM and TG friendly, qualified and low or no cost PrEP service delivery sites exist.

## Supplementary material

To access the supplementary material to this article please see Supplementary Files under Article Tools online.

## Appendix 1. Set of standardized questions used by Adam’s Love online counsellors to assess participants’ risks during the eCounseling session

• age

• ever having an anal intercourse

• number of sexual partners, condom use during anal sex, and HIV testing in the past six months

• date of last possible HIV risk exposure
